# Study of cardiovascular function using a coupled left ventricle and systemic circulation model

**DOI:** 10.1016/j.jbiomech.2016.03.009

**Published:** 2016-08-16

**Authors:** W.W. Chen, H. Gao, X.Y. Luo, N.A. Hill

**Affiliations:** School of Mathematics and Statistics, University of Glasgow, Glasgow G12 8QW, UK

**Keywords:** Cardiovascular modelling, Left ventricle, Systemic circulation, Fluid–structure interaction, Cardio-arterial coupling

## Abstract

To gain insight into cardio-arterial interactions, a coupled left ventricle-systemic artery (LV–SA) model is developed that incorporates a three-dimensional finite-strain left ventricle (LV), and a physiologically-based one-dimensional model for the systemic arteries (SA). The coupling of the LV model and the SA model is achieved by matching the pressure and the flow rate at the aortic root, i.e. the SA model feeds back the pressure as a boundary condition to the LV model, and the aortic flow rate from the LV model is used as the input for the SA model. The governing equations of the coupled system are solved using a combined immersed-boundary finite-element (IB/FE) method and a Lax–Wendroff scheme. A baseline case using physiological measurements of healthy subjects, and four exemplar cases based on different physiological and pathological scenarios are studied using the LV–SA model. The results of the baseline case agree well with published experimental data. The four exemplar cases predict varied pathological responses of the cardiovascular system, which are also supported by clinical observations. The new model can be used to gain insight into cardio-arterial interactions across a range of clinical applications.

## 1 Introduction

Understanding the interaction between the heart and arteries can provide valuable information for clinical diagnosis and treatment in cardiovascular disease (Cecelja and Chowienczyk, 2012, Chirinos, 2013, Ky et al., 2013). However, current mathematical approaches tend to focus on either the arteries (Müller and Toro, 2014, Qureshi et al., 2014), or the heart alone (Perktold and Rappitsch, 1995, Gerbeau et al., 2005). In the former, ventricular function is either prescribed (Urquiza et al., 2006, Olufsen et al., 2000, Figueroa et al., 2006), or simplified using lumped-parameters (Kim et al., 2009, Arts et al., 2005). In the latter, a lumped systemic circulation model is usually used as boundary condition (Žáček and Krause, 1996, Sun et al., 1997, Arts et al., 2005, Lumens et al., 2009, Peskin and McQueen, 1989, Watanabe et al., 2002, Lee et al., 2009, Gao et al., 2014a, Kerckhoffs et al., 2007, Krishnamurthy et al., 2013, Baillargeon et al., 2014).

As changes in arterial properties can alter the heart function and vice versa (Noguchi et al., 2011), in this paper, we focus on the coupling of the heart and the arteries, by combining a models of a 3D left ventricle (LV) with a systemic arteries (SA) model that uses a structural tree description of the vascular beds containing the smaller arteries. The 3D LV is reconstructed from a dataset of in vivo magnetic resonance imaging (MRI) of a healthy volunteer (Gao et al., 2015a), which includes fluid–structure interaction (FSI). The SA model is based on the development by Olufsen (1999) and Olufsen et al. (2000), which includes both large arteries and remote vascular beds.

## 2 Methodology

**Fig. 1:**
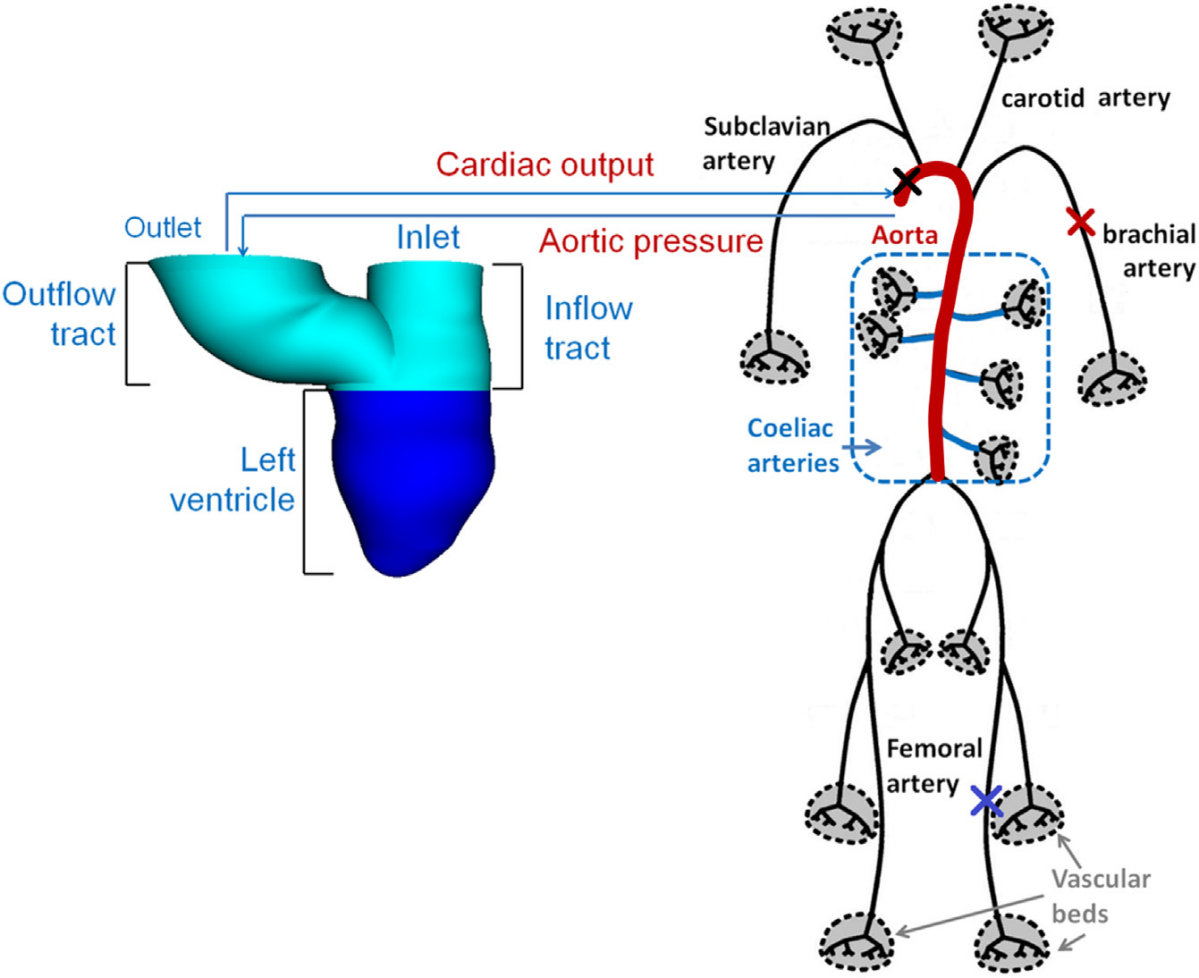
Schematic illustration of the coupled 3D LV and the 1-D systemic circulation model. The 3D LV is immersed in a 16.5cm×16.5cm×16.5cm fluid box, and the LV is meshed with 138k tetrahedral elements. Pressure and flow rate are obtained in the LV and at midpoints of each vessel in three groups of the large arteries, i.e. aorta (red), coeliac arteries (black), and other long arteries (black). ‘×’ denotes the midpoints of the ascending aorta (thicker curve), brachial, and femoral arteries.

The coupled left ventricle and the systemic artery (LV–SA) model is shown in Fig. 1. The methodologies for the 3D LV and the 1D SA models have been published elsewhere (Gao et al., 2014a, Olufsen et al., 2000, Olufsen et al., 2012), but are briefly described here to explain the coupling procedure.

### 2.1 The LV model

The LV model consists of the valvular and inflow/outflow tracts (assumed passive), and the active LV region. The model is solved using a combined immersed boundary finite element (IB/FE) method. Let $\Omega \subset R^{3}$ denote the physical domain occupied by the fluid–structure interaction (FSI) system, in which $x=(x_{1},x_{2},x_{3})\in \Omega$ are fixed Eulerian coordinates. Let $U\subset R^{3}$ denote the reference configuration of the immersed solid, in which $X=(X_{1},X_{2},X_{3})\in U$ are Lagrangian coordinates. χ(X,t) describes the physical position of the material point **X** at time *t*, and then the physical region occupied by the immersed solid at time *t* is $\chi (U,t)=\Omega_{s}(t)\in \Omega$, and the region occupied by the fluid at time *t* is $\Omega_{f}(t)=\Omega ⧹\Omega_{s}(t)$ (‘⧹’ is the setminus operator). The formulation of the FSI equations is (Griffith, 2012, Gao et al., 2014b)

(1) $$\rho (\frac{\partial u}{\partial t}(x,t)+u(x,t)\cdot \nabla u(x,t))=-\nabla p(x,t)+\mu \nabla^{2}u(x,t)+f^{s}(x,t),$$

(2) ∇·u(x,t)=0,

(3) $$\frac{\partial \chi }{\partial t}(X,t)=\int_{\Omega }u(x,t)\delta (x-\chi (X,t))\;dx,$$

(4) $$f^{s}(x,t)=\int_{U}\nabla_{X}\cdot P^{s}(X,t)\delta (x-\chi (X,t))\;dX-\int_{\partial U}P^{s}(X,t)\cdot N(X)\delta (x-\chi (X,t))\;dA,$$

in which *μ* is the fluid viscosity, u(x,t) is the fluid velocity of the blood, and p(x,t) is the pressure, N(X) is the exterior unit normal to *U*, and δ(x) is the three-dimensional Dirac delta function. We assume that the fluid and the solid have the same density *ρ*. $P^{s}(X,t)=\det(F)\sigma^{s}F^{-T}$ is the first Piola–Kirchhoff stress tensor, and $\sigma^{s}$ is the structure Cauchy stress tensor,

(5) $$\sigma^{s}=\sigma^{p}+\sigma^{a},$$

where $\sigma^{a}=T_{0}\;T(f⊗f)$ is the active stress, while the contractile tension *T* is described by the myofilament model of Niederer et al. (2006), which is triggered by a prescribed intracellular calcium transit (Hunter et al., 1998), as shown in Fig. 3. *T*_0_ is introduced to make the contraction patient-specific. The passive stress $\sigma^{p}$ is determined through the Holzapfel–Ogden strain energy function (Holzapfel and Ogden, 2009), as detailed in the Appendix A.

Equations (1), (2) are discretized using a finite-difference method, and Eqs. (3), (4) are discretized with a finite-element method. The material parameters in (12) are determined inversely by fitting the measured end-diastolic volume and myocardial strains using a multi-step optimization procedure (Gao et al., 2015b). *T*_0_ is determined by matching the measured stroke volume (Gao et al., 2014a). The valvular region is modelled as a neo-Hookean material, with the shear modulus adjusted so that its maximum displacement agrees with MRI measurements. The inflow/outflow tracts are both assumed to be rigid, with the inlet and outlet annuli fixed in space.

### 2.2 The systemic arterial model

The SA model consists of 24 large arteries modelled as a one-dimensional cross-sectional-area-averaged flow and pressure. Each terminal vessel in the network of the large arteries is coupled with a group of small arteries (the vascular bed), which are modelled as an asymmetric structured-tree to provide outflow boundary conditions (Olufsen, 1999, Olufsen et al., 2000, Olufsen et al., 2012).

The governing equations for the SA model are

(6) $$\frac{\partial Q}{\partial x}+\frac{\partial A}{\partial t}=0,$$

(7) $$\frac{\partial Q}{\partial t}+\frac{\partial }{\partial x}(\frac{Q^{2}}{A})+\frac{A}{\rho }\frac{\partial P}{\partial x}=-\frac{2\pi \nu R}{\delta^{⁎}}\frac{Q}{A},$$

(8) $$P(x,t)-P_{0}=\frac{4}{3}\frac{\mathrm{Eh}}{r_{0}}(1-\sqrt{\frac{A_{0}}{A}}),$$

where *Q* is the volumetric flow rate, *A* is the cross-sectional area, *P* is the averaged cross-sectional pressure, *P*_0_ is the constant external pressure, *ρ* is the density, *ν* is the kinematic viscosity, *R* is the radius of the vessel. *A*_0_ and *r*_0_ are the cross sectional area when $P=P_{0}$, $\delta^{⁎}$ is the width of the boundary layer ($\delta^{⁎}⪡R$), *h* is the wall thickness, and *E* is the arterial Young׳s modulus, computed as,

(9) $$\frac{\mathrm{Eh}}{r_{0}}=k_{1}\;\exp(k_{2}\;r_{0})+k_{3},$$

where *k*_*i*_ (*i*=1–3) are material constants (Olufsen et al., 2000). These equations are solved numerically using the Lax–Wendroff scheme (Lax and Wendroff, 1960). With each vascular bed, each parent artery with radius *r*_*p*_ divides into two daughter arteries with radius $r_{d_{1}}=\alpha r_{p}$ and $r_{d_{2}}=\beta r_{p}$, (0<β<α<1). The bifurcation process continues until the radius of the daughter vessel reaches the minimum radius $r_{\min}=100\;\mu m$. The radius exponent *ξ*, the asymmetry ratio *η*, and the area ratio *γ* (only two of which are independent) are given by

(10) $$r_{p}^{\xi }=r_{d_{1}}^{\xi }+r_{d_{2}}^{\xi },\;2.33\leq \xi \leq 3,$$

(11) $$\gamma ={\left(\frac{r_{d_{1}}}{r_{d_{2}}}\right)}^{2},\;\eta =\frac{r_{d_{1}}^{2}+r_{d_{2}}^{2}}{r_{p}^{2}}=\frac{1+\gamma }{{\left(1+\gamma^{\xi /2}\right)}^{2/\xi }},\;\eta >1.$$

### 2.3 Coupling of the SA model and the LV model

**Fig. 2:**
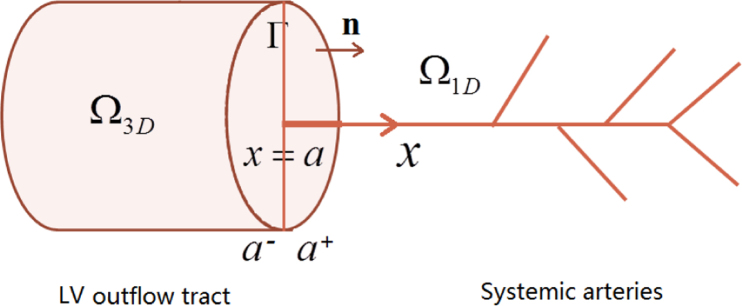
The interfacial plane *x*=*a* at the location of the aortic valve (AV), which connects the 3D LV model to the 1D SA model. *Γ* is the interface between the LV and SA models, ‘−’ means in the LV model, ‘+’ means in the SA model. The coupling conditions of the two models are detailed in Appendix C.

The coupling is achieved by matching the pressure ($P_{-}$) and the flow rate ($Q_{-}$) at the outflow tract (corresponding to the beginning of the ascending aorta) of the 3D LV model to these of 1D SA model ($P_{+}$ and $Q_{+}$) at interface plane *Γ*_*a*_ (Fig. 2). Subscripts ‘+’ and ‘−’ are used for representing variables in the SA and the LV models, respectively. Before coupling, the SA model is initialized for four periods using a prescribed cardiac output from a decoupled LV model (Gao et al., 2015a). The parameters of the baseline case are summarized in Appendix B.

Since the LV and SA models are coupled only in systole when the aortic valve (AV) is open, a set of simplified boundary conditions are used in our coupled model as detailed in Appendix C. In the SA model, the time step is $\Delta t_{\mathrm{SA}}=t_{\mathrm{period}}/N=0.9/8192\approx 1.10\times 10^{-4}\;s$, in which $t_{\mathrm{period}}$ is the length of period and *N* is the number of time steps during one period. In the LV model, a basic time step $\Delta t_{\mathrm{LV}}=1.22\times 10^{-4}\;s$ is used in the diastolic and relaxation phases, but smaller time steps are used in systole ($0.125\Delta t_{\mathrm{LV}}$) as much higher structural stress is generated from the myocardial contraction. Interpolation is used to ensure the pressure and the flow rate are exchanged at the right time points when the two models are coupled. Simulation of the 3D LV model is implemented using the open-source IBAMR framework (https://github.com/IBAMR/IBAMR); the 1D SA model and the interactions are implemented using C++ within the same framework. The LV–SA model converges to periodic solutions in the second period after coupling, and the maximum pressure difference between the 2nd and the 3rd periods is less than 1%. Hence the results from the second period are presented. The simulation time for a cardiac cycle is around 168 h (7 days) on a local Linux workstation with eight Intel(R) Xeon(R) CPU cores (2.65 GHz) and 32 GB RAM.

### 2.4 Cases different to the baseline

We also consider the following pathological scenarios.

• *Case* 1 — *Stiffening of the arterial wall*: Increased arterial stiffness is associated with an increased risk of cardiovascular events such as myocardial infarction and stroke (Lee and Oh, 2010, Avolio et al., 1983). Here we increase *k*_3_ in (9) by 100% in the large arteries to investigate how arterial stiffness affects the LV function.
• *Case* 2 — *Vascular bed rarefaction*: Rarefaction, a reduction in the density of small arterioles, is often associated with Type II diabetes (Hopkins and McLoughlin, 2002), and has a significant impact on cardiovascular system (Olufsen et al., 2012, Safar et al., 2003). Rarefaction is simulated by decreasing the radius exponent *ξ* in (10) from 2.76 to 2.4, thus reducing the total number of small vessels by 71% compared to the baseline case.
• *Case* 3 — *Decreased compliance*: LV hypertrophy or fibrosis is usually linked with a stiffer myocardium (Beckett et al., 2008). We increase the myocardium stiffness by doubling the values of *a*, $a_{f}$, $a_{s}$ and $a_{\mathrm{fs}}$ in Eq. (12) in Appendix A.
• *Case* 4 — *Increasing the myocardial contractility*: Myocardial contractility, or isotropy, is one of the three primary mechanisms that regulate the LV stroke volume. To model the effects of increased LV contractility, we increase *T*_0_ in (5) by 33% from the baseline case.
• *Cases* 1_*iso and* 2_*iso*: We also apply Cases 1 and 2 to an isolated SA model as Case 1_iso and Case 2_iso, respectively, to identify coupling effects.

## 3 Results

### 3.1 The baseline case

**Fig. 3:**
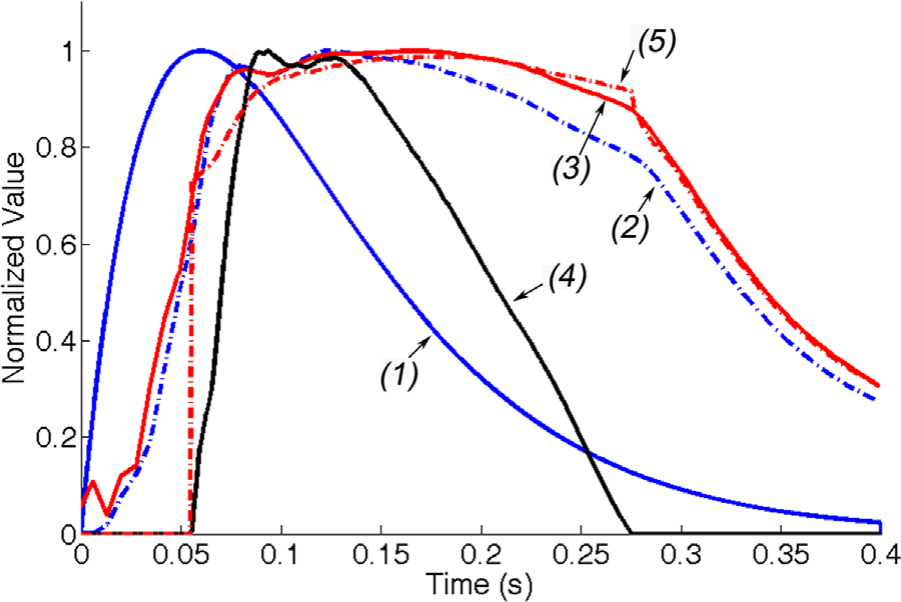
Results of the baseline case in systole for a prescribed calcium transit (1), peak value=10^−7^ μmol. All results are normalized with their respective peak values, except for the pressure at outflow tract, which is normalized with the peak LV center cavity pressure. The average active stress (2) has peak value=68.9 kPa, the LV center cavity pressure (3) has peak value=113.1 mmHg, the aortic flow rate (4) has peak value=532 mL s^−1^, and the pressure at the outflow tract (5) has peak value=111.7 mmHg. Time *t*=0 indicates the beginning of the isovolumetric contraction.

The results of the coupled model in systole for the baseline case are plotted in Fig. 3; the intracellular calcium transient taken from Hunter et al. (1998) is also shown for comparison. The corresponding clinical indices are summarized in Table 1. The ejection fraction (EF) is 51%. The pressure curve closely follows the active stress, with a small delay behind the intracellular calcium transient.

**Fig. 4:**
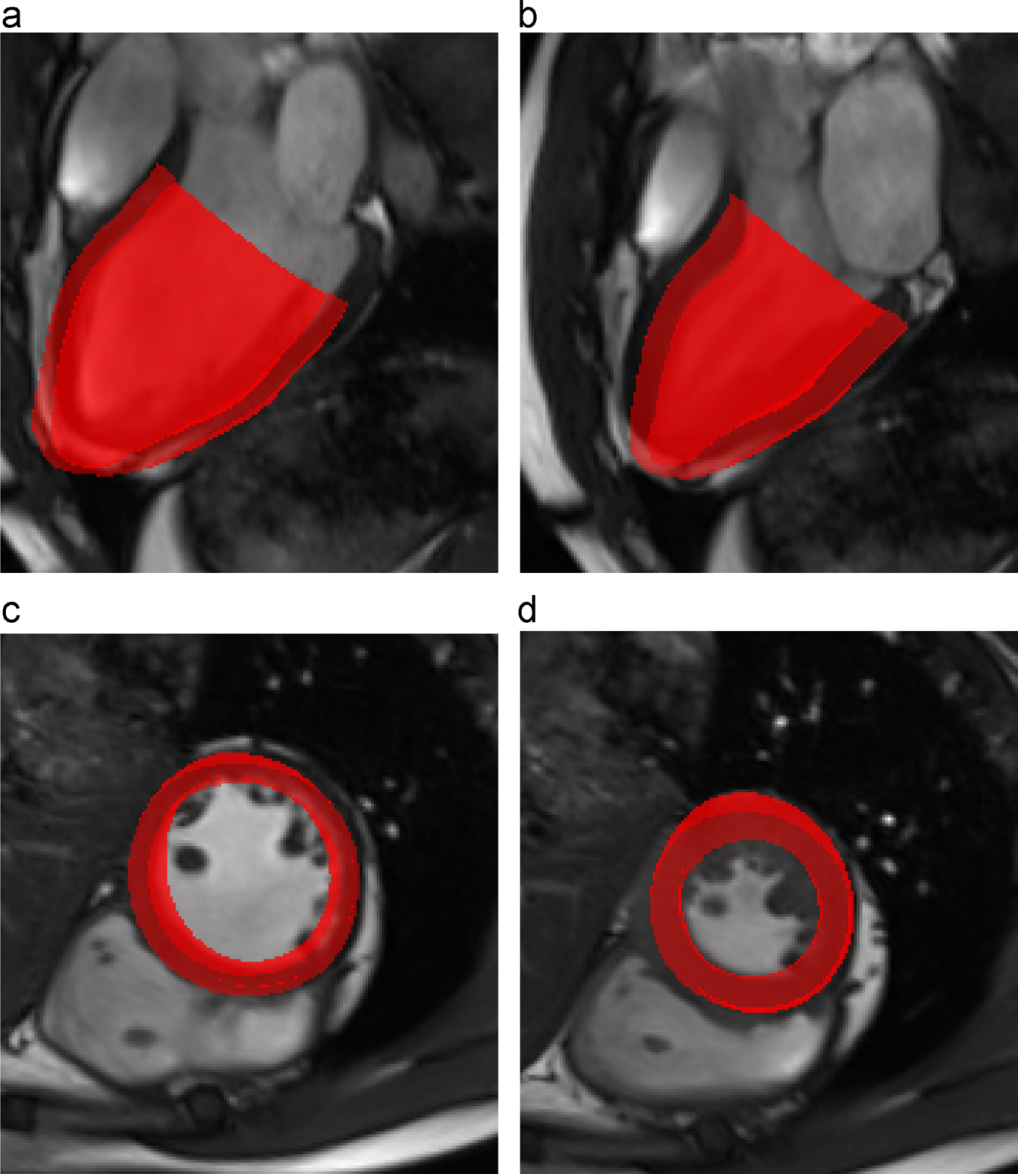
The computed 3D LV geometry at end-diastole (a) and (c), and end-systole (b) and (d), superimposed with the long-axis and short-axis cine MR images. Notice that in (c) and (d) the computed geometry is in 3D (with the apex chopped off), hence the comparison appears to be worse in places when overlapped with the 2D MR images, since LV is not entirely cylindrical. The overall agreement is very good.

**Fig. 5:**
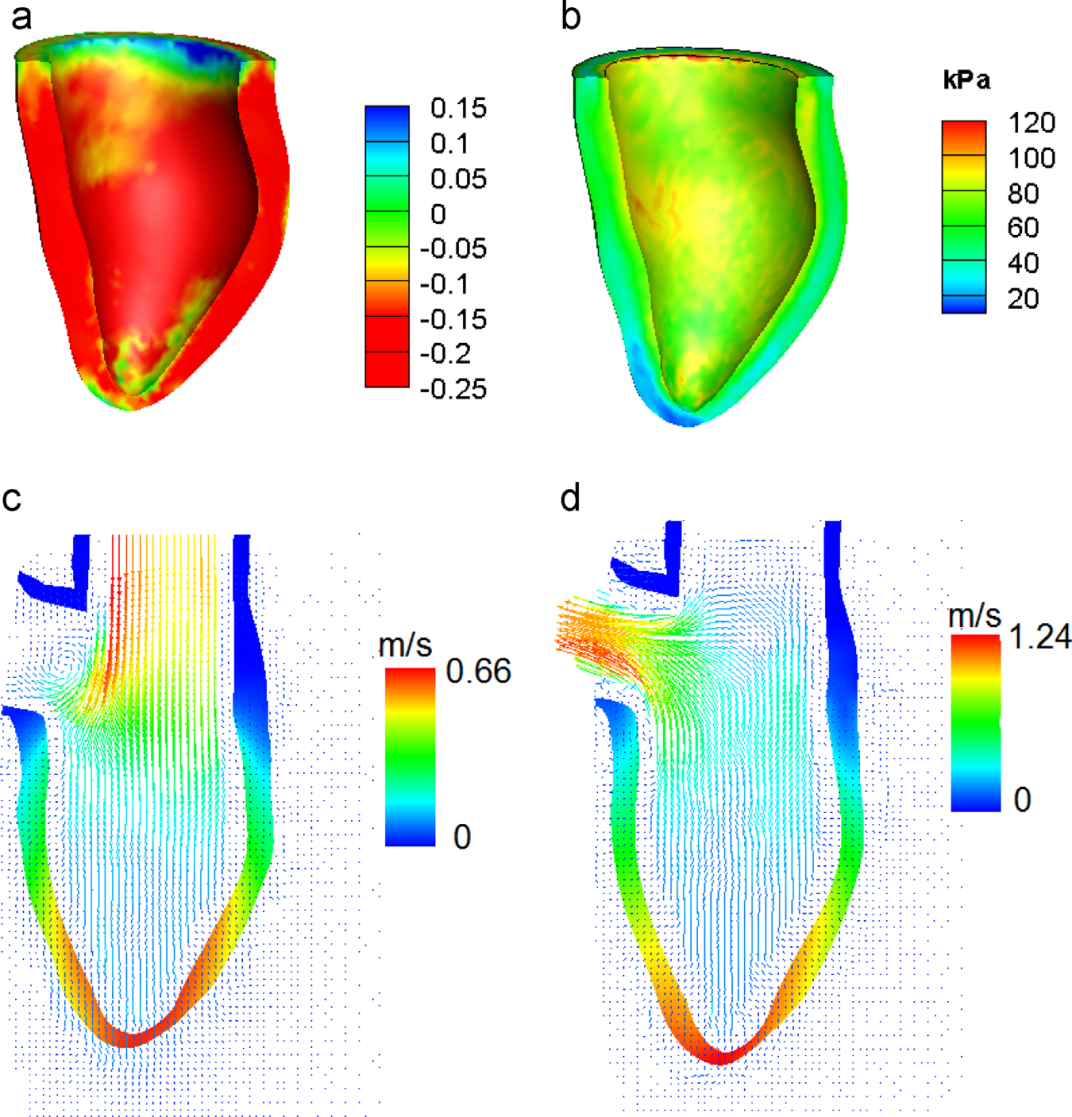
The computed fields for the Baseline case: (a) the myofibre strain at end-systole (mean value=−0.17), (b) the active stress at mid-systole (mean value=66.5 kPa), and the flow in the central plane in (c) mid-diastole, and (d) mid-systole. We note the active strain/stress near the top plane is not very accurate due to the assumption that the tracts are non-contractile.

The deformed LV geometry (in red) at end-diastole and end-systole are shown in Fig. 4(a–d), superimposed on the corresponding cine MR images. Qualitative assessment by visual inspection suggests that the computed LV geometries agree well with the MR images in most parts of the LV, with exception of the apex, where our model overshoots slightly. This is presumably due to the fact that our model does not include the pericardium, which would constrain the apical motion. The myofibre strain field $\left(f\cdot \frac{{\mathrm{FF}}^{T}-I}{2}\;f\right)$ at end-systole is shown in Fig. 5(a). The mean value of the strain is −0.17, which is comparable to previously reported systolic strain values (Moore et al., 2000). Figure 5(b) shows the active stress ($\sigma^{a}$) during mid-systole. All regions appear to contract equally hard with the exception of the apical region. Flow patterns at mid-diastole and mid-systole are shown in Fig. 5(c) and (d) respectively. Since there is no mitral valve, the flow vortex in Fig. 5(c) is not as distinct as when the mitral valve is present (Yin et al., 2010). This inaccuracy in the flow fields may not significantly affect the overall results as it is commonly agreed that blood flow affects the LV deformation mostly through the pressure.

**Fig. 6:**
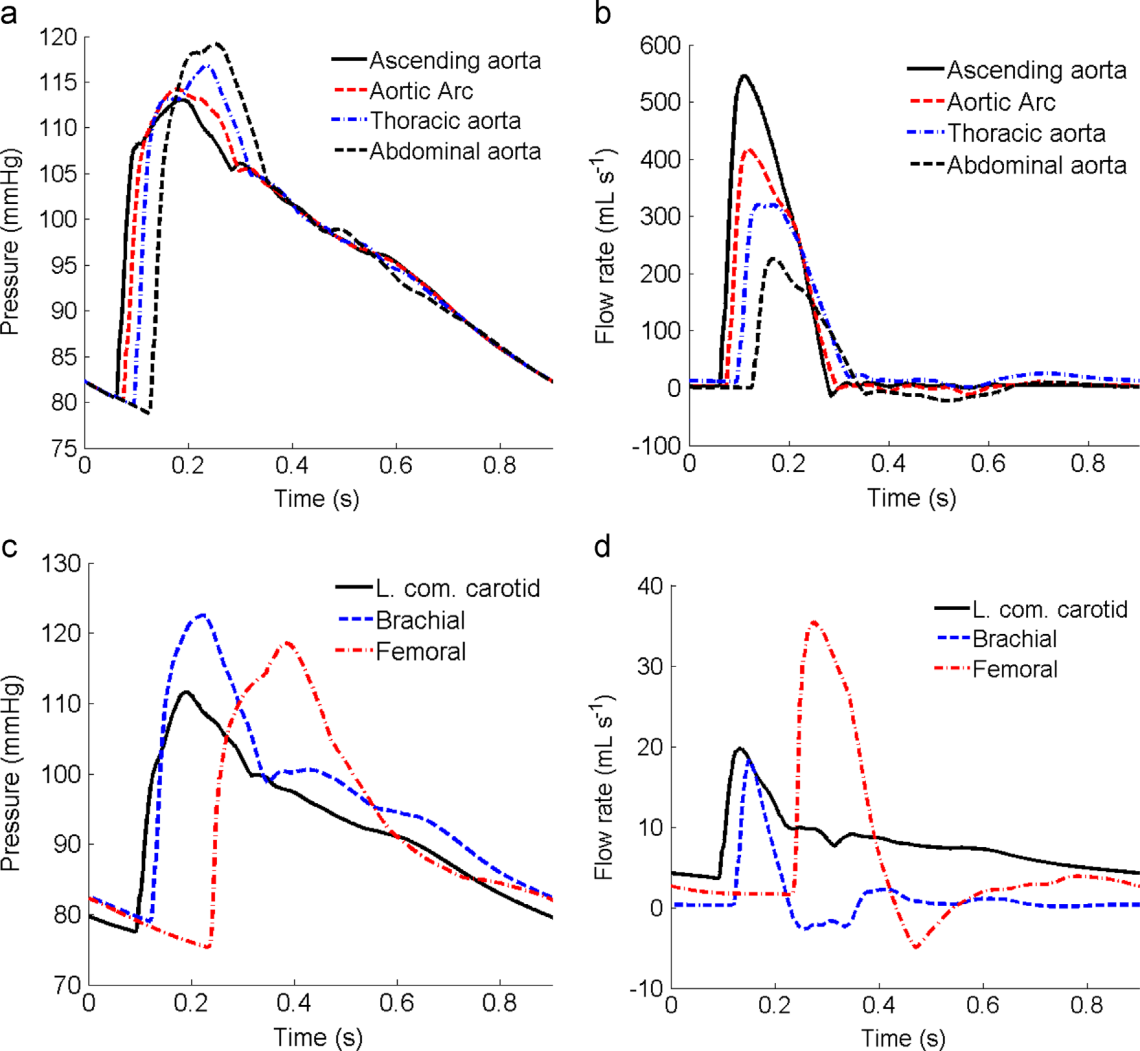
The simulated pressure along, (a) the aorta, and (c) the left common carotid, brachial and femoral arteries, for the baseline case over a cardiac cycle. The corresponding flow rates are shown in (b) and (d).

The pressure and flow waveforms in selected arteries are shown in Fig. 6, with the indices summarized in Table 2. A clear trend in the time delay of the peak pressure during systole with distance away from the heart can be seen from Fig. 6(a). At 0.25 s, there is a pronounced dicrotic notch in the ascending aorta due to the reflected flow at end-systole, which fades away in the distal arteries. A similar time delay is present in the flow rate waveform in the distal arteries (Fig. 6(b)); the peak flow rate arrives approximately 64 ms earlier in the ascending aorta than that in the abdominal artery. Figure 6(c) and (d) plots the pressure and flow rate waveforms in the long arteries such as the carotid, brachial and femoral arteries. The systolic (126 mmHg) and diastolic (81 mmHg) pressures in the mid-brachial artery are within the normal range (Sesso et al., 2000).

In summary, the results from both the LV and the systemic circulation in the baseline case lie within a normal physiological range (Starling, 1993, Moore et al., 2000, Sesso et al., 2000).

### 3.2 Cases 1–4

**Fig. 7:**
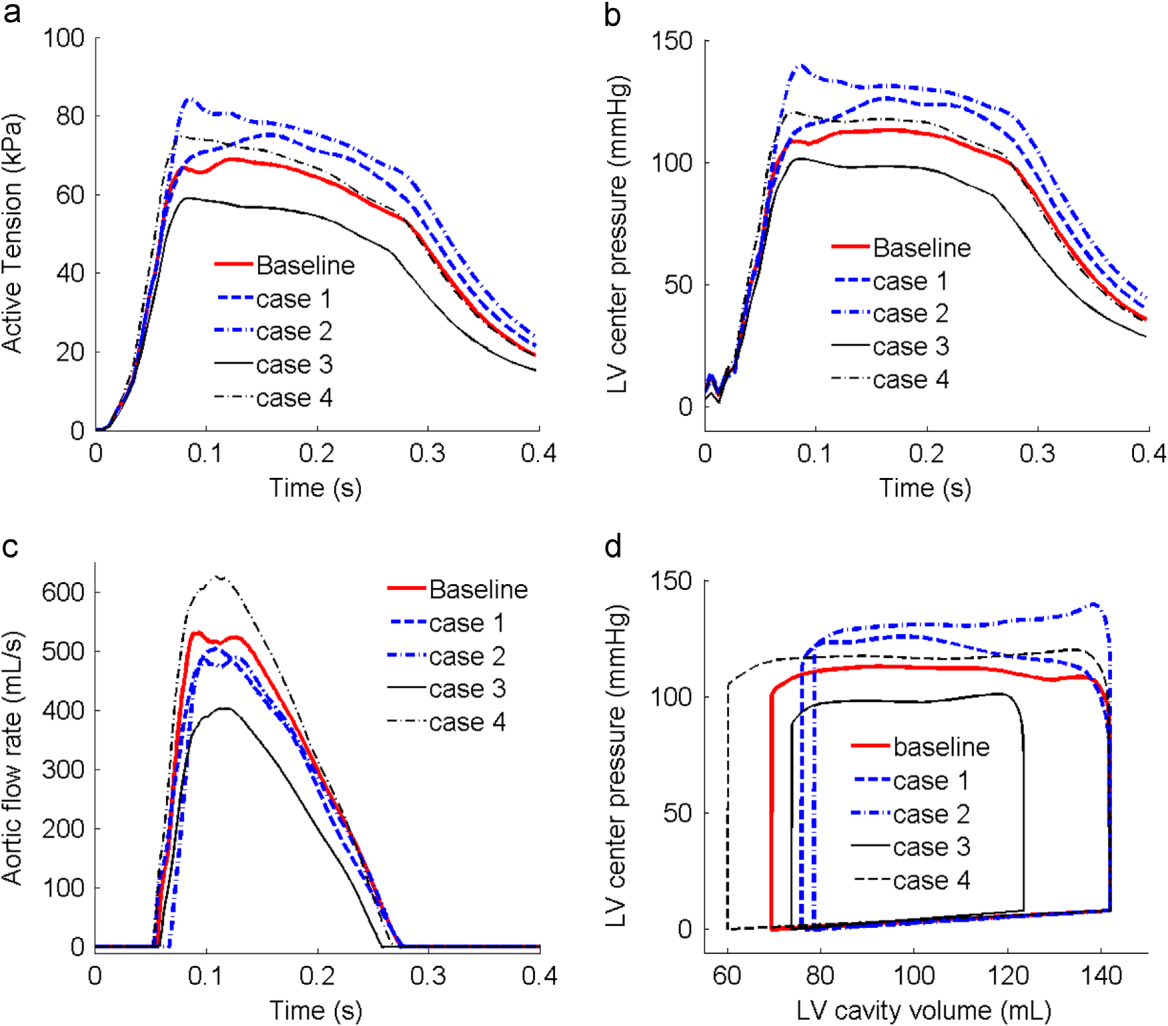
The computed (a) active tension, (b) LV center cavity pressure, (c) aortic flow rate, and (d) pressure–volume loop, for all the cases simulated over a cardiac cycle.

The results of the active stress, LV pressure and the aortic flow rates from the coupled model in systole are compared for all the cases in Fig. 7. We can see that stiffened arteries (Case 1) result in a higher ventricular pressure, and a reduced aortic flow rate. Rarefaction (Case 2) causes a similar flow rate reduction as in Case 1, but has the highest increase in pressure and active stress. Decreased LV compliance (Case 3) leads to a much smaller end-diastolic volume. As the active stress is a function of strain, a smaller end-diastolic volume indicates a small expansion of the LV, hence both the active stress and LV pressure decrease, leading to a lower aortic flow rate and shorter systolic ejection duration. Increased contractility (Case 4) leads to a moderate pressure rise compared to the baseline case, but causes the highest flow rate. Interestingly, Case 4 does not yield the highest active stress (Table 1).

The pressure–volume loops are shown in Fig. 7(d). Cases 1–3 all lead to a reduced stroke volume, with Case 3 (stiffener LV) being the most severe. The decreased compliance (Case 3) has the most notable reduction in the stroke work (indicated by the enclosed area of the pressure–volume loop), while increased contractility (Case 4) is associated with the largest stroke work. The indices of the LV pump function are summarized in Table 1. Case 3 (stiffer LV) has the worst pump function indices, with stroke volume reduced by 31%, and significant drops in the LV pressure and active tension. Compared to the baseline case, there is an increase in the peak active tension and LV pressure, and a smaller decrease in the stroke volume in Cases 1 and 2, although the stroke work is higher in Case 2, and lower in Case 1. Both the stroke volume and the stroke work are significantly increased in Case 4 due to the faster ejection speed.

**Fig. 8:**
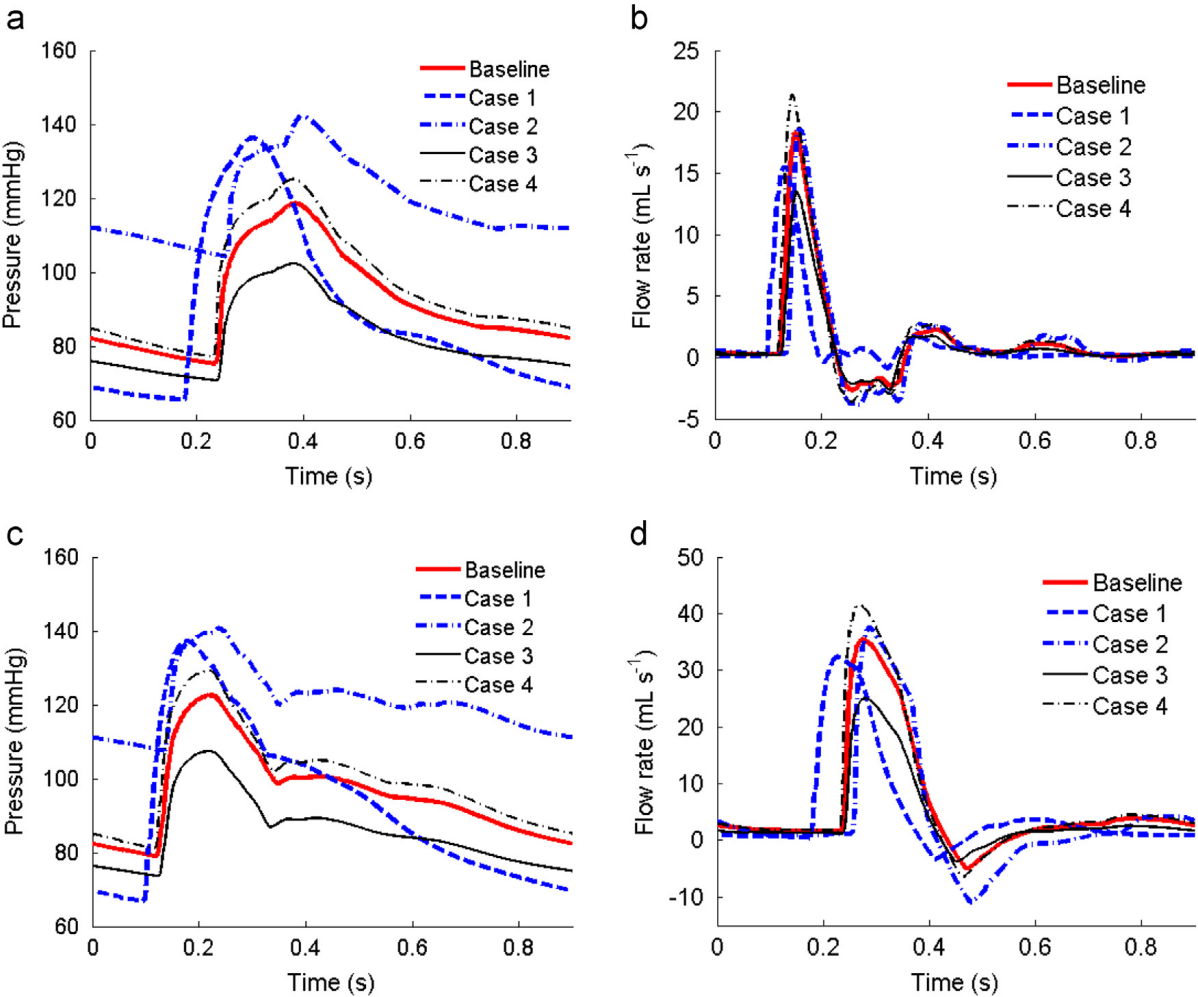
Comparison of the predicted pressure (left) and flow rate (right) profiles in the mid-brachial (a, b), and mid-femoral artery (c, d), for all the cases over a cardiac cycle. The results are taken from the locations marked with ‘×’ in Fig. 1.

The changes in the LV are carried through to the systemic arteries, as shown in Fig. 8. The key indices of the SA system are summarized in Tables 2 and 3 for selected arteries.

### 3.3 Case 1_iso and Case 2_iso

To identify LV effects on the systemic circulation in pathological situations, we also compare the results from the isolated systemic circulation model with these of the coupled model for Cases 1 and 2. As expected, the general trends in the pressure and flow rate waveforms are similar in both cases. However the isolated SA model tends to over-estimate the results by up to 7% for pressure, and up to 20% for flow rate, as shown in Table 4.

## 4 Discussion

Our coupled model makes it possible to study more detailed interactions between the LV and the systemic arteries under various normal and pathological conditions during systole. Although the LV and the systemic models were initially derived from different healthy human subjects, through inverse parameter estimation, we are able to match the measurements of the LV subject according to the end-diastolic and stroke volumes for the baseline case. All of the computed indices are in line with the previously reported values (Sesso et al., 2000, Moore et al., 2000, Mahadevan et al., 2008).

The coupled model is used to examine four different scenarios. Cases 1 and 2 show how the LV reacts to an increased LV afterload due to changes in the systemic circulation. Cases 3 and 4 show the impact of the changed LV function on the systemic circulation. For both Cases 1 and 2, the LV needs to generate a higher active tension and pressure, yet still suffers from a reduced stroke volume. This could promote adverse remodelling of the LV in the longer term. Rarefaction also increases both the peak and mean pressures in the large arteries, which agrees with clinical observations that rarefaction in vascular beds can lead to hypertension (Noon et al., 1997, He et al., 2010, Antonios, 2006). In Case 3, the stiffer LV represents a potential heart disease, post-myocardial fibrosis. This scenario gives the most markedly reduced pumping performance: the stroke volume, active tension, and LV pressure are all significantly decreased. With increased *T*_0_ (Case 4), an immediate effect is the increased stroke volume (by 13%). This suggests that during certain conditions, myocardium could remodel itself to enhance contractility in order to meet the flow demand (Zhang et al., 2010). However increased myocardial stress in this case may further induce myocyte hypertrophy and a consequent increase in myocyte death rate (Gomez et al., 2001, Abbate et al., 2003).

**Fig. 9:**
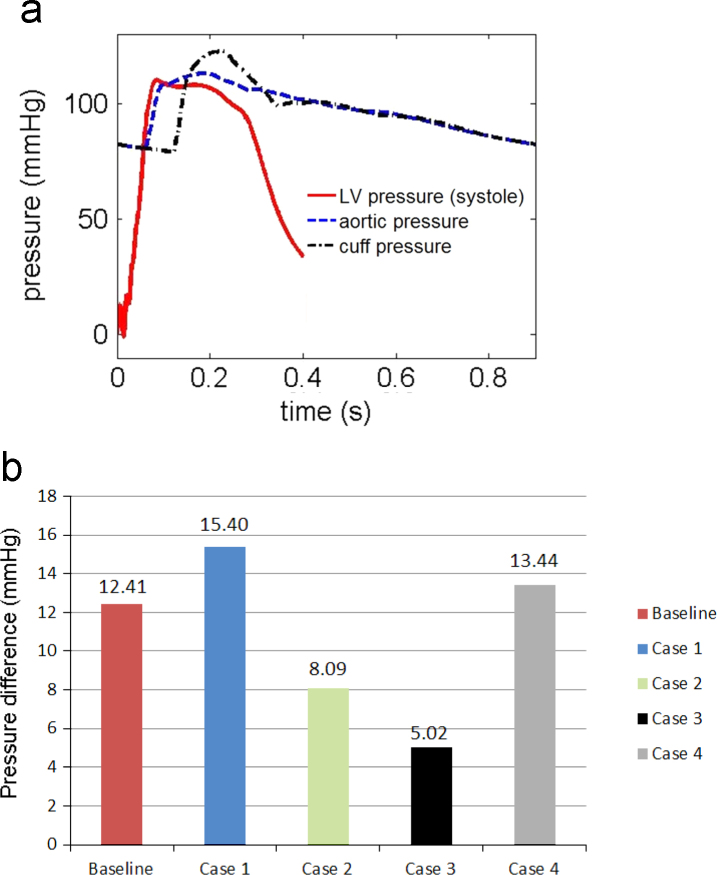
(a) The predicted left ventricular/aortic pressures vs cuff pressure for the baseline case, and (b) comparison of the pressure difference from the LV to station “X” at the brachial artery as marked in Fig. 1, for all the simulated cases.

Cuff (brachial) pressure is routinely measured by clinicians as an indication of the central (ascending aorta) pressure and the pressure inside LV. However, the difference between the cuff and the central pressures in vivo is unclear. Our model shows that the pressure drop between the LV and the ascending aorta is minor, see Fig. 9(a), and the pressure difference between the brachial artery and the center of the LV for the baseline case is about 12.41 mmHg. This agrees with Kroeker and Wood (1955) who observed that, although mean and diastolic blood pressure are relatively constant throughout the arterial tree, there is a gradual increase in systolic pressure moving from the aorta to the peripheral arteries. Indeed, the pressure measured at the brachial artery can be 5–20 mmHg higher than that in the ascending aorta. This pressure amplification arises principally because of increased vessel stiffness and changes in vessel geometries. Our results also suggest that this pressure difference varies, as shown in Fig. 9(b). Stiffer arteries (Case 1) and increased contractility (Case 4) cause the greater pressure amplifications, while the difference is less in rarefaction (Case 2). The most prominant pressure amplification is seen in Case 3 (stiffer LV).

**Fig. 10:**
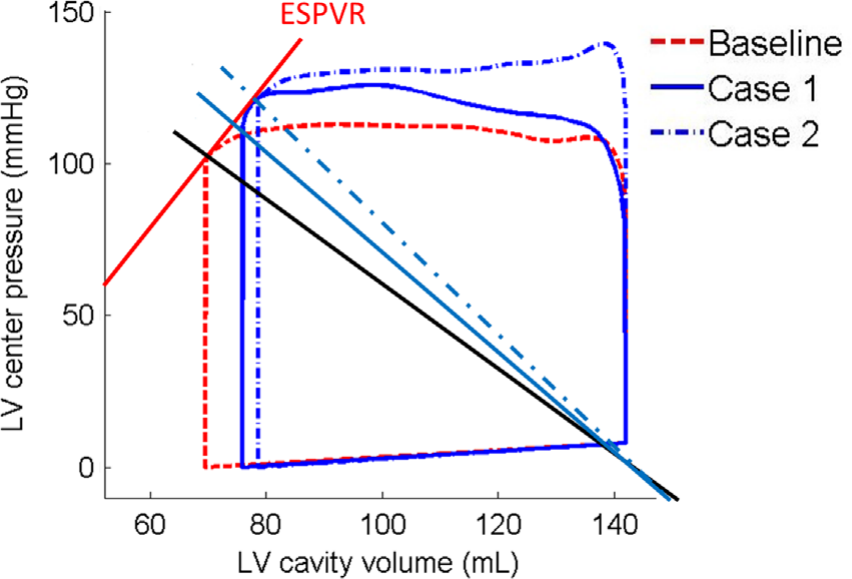
Derivation of the effective arterial elastance (*E*_*A*_) and LV end-systolic elastance (*E*_*S*_, the slope of the ESPVR or end-systolic pressure–volume relation) based on the P–V loops for the Baseline case, and Cases 1 and 2.

We can show how the arterial elastance (*E*_*A*_, the ratio of end-systolic LV pressure to stroke volume) changes the shape of the P–V loop and the end-of-systolic P–V relationship (ESPVR) in Fig. 10. *E*_*A*_ can be increased either by stiffer arteries (Case 1) or rarefaction (Case 2). The interaction between the LV and the systemic circulation can also be assessed by the LV chamber elastance (*E*_*S*_), the slope of ESPVR (Sunagawa et al., 1983, Chirinos, 2013), and by the ratio $E_{A}/E_{S}$. Clinically, *E*_*S*_ can be obtained by producing step-wise pharmacological afterload variations without inducing inotropic changes. In the simulated Cases 1 and 2, the afterload for the coupled LV model (aortic pressure) is increased, but the contractility is not, therefore we can obtain *E*_*S*_ by fitting the three end-systolic points (Cases 1, 2 and baseline) at the P–V curves (Fig. 10). The ratio $E_{A}/E_{S}$ in the baseline case is 0.72, which lies in the reported range for normal humans (0.62–0.82) (Redfield et al., 2005). In fact, it is close to the value when the myocardial energetic efficiency is maximized (0.7) (De Tombe et al., 1993). $E_{A}/E_{S}$ is 0.87 and 0.95 for Cases 1 and 2, suggesting that the coupling between the LV and systemic circulation is sub-optimal. Our model predictions are consistent with clinical interpretations of how *E*_*A*_ and *E*_*s*_ change the P–V curve, but provide more quantitative information on exactly what causes such changes. Such a coupled model could be used together with measurable values (such as cuff pressure), to give greater clinical insight.

Comparison between the results of the coupled model and that of the isolated circulation model suggests that the isolated circulation model overestimates the peak pressure and the flow rate. This is an example of how the circulation system is affected by the LV. When either increase in the arterial stiffness or rarefaction, the LV tends to increase the active tension and decrease the cardiac output, which in turn affects the systemic circulation.

We now mention the limitations of the model. The LV and systemic arteries are from two different volunteers. To make the coupled model work physiologically and agree with the measured systolic and diastolic volumes of the LV subject, a number of parameters are tuned, such as the external pressure *p*_0_ in the SA model, the myocardial passive stiffness, and *T*_0_, which controls the magnitude of the active tension. All other parameters in the SA model are kept the same as in Olufsen et al. (2000). Therefore the coupled model is not patient-specific per se. In our model of the systemic arteries, the pulse wave velocity (PWV) is determined by the pressure–area relationship for the arteries (the tube law), specifically $c(P)=\sqrt{\left(A/\rho )(\partial P/\partial A\right)}$. We have studied the clinical estimates of the PWV in a model of the pulmonary circulation (Qureshi and Hill, 2015). It would be possible in principle to use the clinical estimates of the PWV to assess the tube law although errors in the measurements may make this difficult. However, it is probably even more interesting to use wave intensity analysis to identify waves reflected from bifurcations in the arterial system and to study their contribution to the pressure loading on the LV during systole in health and disease. This work is underway.

Throughout the study, we have kept the end-diastole pressure at 8 mmHg, although this value is known to change in pathological conditions. In addition, we use velocity boundary conditions to mimic the action of the AV, thus the pressure drop across the AV is not accounted for. The LV and the systemic arteries coupling only occurs during systole (from end-diastole to end-systole) because when the AV is closed, the flow in the arterial system is separated from the LV. Moreover, the LV diastolic filling is simplified by not including the mitral valve, the left atrium and the pulmonary circulation. While our coupled model can address a range of clinical questions as we have illustrated here, more involved pathological problems may require the development of more sophisticated models of cardiovascular system that include the four chambers of the heart, as well as the pulmonary circulation.

## 5 Conclusions

We have developed a coupled model for studying the interaction between a three-dimensional contracting left ventricle model and a structured-tree based cross-sectional-area-averaged systematic circulation model. The coupled model can predict details of the left ventricular dynamics, pressure and flow rate profiles at any position in the systemic arteries throughout the cardiac cycles, and thus it provides a powerful in silico tool for exploring and understanding cardio-arterial interactions. This new model is used to study a number of pathological changes in the left ventricle and the systemic circulation. The results from the coupled model are consistent with clinical observations. We find that stiffening of the arterial wall and functional rarefaction in the remote vascular beds cause higher blood pressure along with higher LV active tension, but with reduced stroke volume. A stiffer LV leads to severely impaired pump function with low active tension, stroke volume and low blood pressure. Increased contractility can help the heart to maintain a higher stroke volume, but gives rise to an elevated pressure in the circulation. Furthermore, the model can be combined with clinical measurements, such as cuff pressure, to infer pressure profiles inside the LV. With further development towards patient-specific individualization, the model can be applied to a range of clinical studies for exploring the causes and development of cardiovascular diseases and their potential treatments.

## Conflict of interest

None declared.

## Figures and Tables

**Table 1:** Comparison of systolic LV pump functions of the baseline and Cases 1–4. All changes for Cases 1–4 are in percentage compared to the baseline case.

Indices	$T_{\mathrm{peak}}$	$P_{\mathrm{peak}-\mathrm{ao}}$	$P_{\mathrm{peak}-\mathrm{LV}}$	$Q_{\mathrm{peak}-\mathrm{ao}}$	SV	SW	$t_{\mathrm{ej}}$
**Baseline**	**68.9**	**111.7**	**113.1**	**532.3**	**72.6**	**102.9**	**220**
Units	kPa	mmHg	mmHg	mL s^−1^	mL	cJ	ms
Case 1 (stiffer arteries) (%)	+9.1	+11.8	+11.4	−5.2	−8.8	−0.9	+2.3
Case 2 (rarefaction) (%)	+22.2	+18.4	+23.4	−8.0	−12.5	+4.9	−5.0
Case 3 (stiffer LV) (%)	−14.4	−11.9	−10.7	−24.3	−31.4	−39.4	−8.6
Case 4 (contractivity) (%)	+8.4	+3.9	+6.1	+17.8	+13.0	+19.4	−1.8

**Table 2:** Summary of the indices of the pressure waveform: the peak arterial blood pressure ($P_{\mathrm{peak}}$), the time when pressure peaks ($t_{\mathrm{peak}}$), and the arterial trough pressure ($P_{\mathrm{trough}}$), recorded at mid-ascending aorta, mid-femoral and mid-brachial arteries.

Position	Mid-ascending aorta			Mid-femoral			Mid-brachial		
Indices	$t_{\mathrm{peak}}$	$P_{\mathrm{peak}}$	$P_{\mathrm{trough}}$	$t_{\mathrm{peak}}$	$P_{\mathrm{peak}}$	$P_{\mathrm{trough}}$	$t_{\mathrm{peak}}$	$P_{\mathrm{peak}}$	$P_{\mathrm{trough}}$
Unit	s	mmHg	mmHg	s	mmHg	mmHg	s	mmHg	mmHg
**Baseline**	**0.170**	**113**	**81**	**0.368**	**119**	**80**	**0.205**	**126**	**81**
Case 1 (%)	+12.9	+12.4	−16.0	−20.9	+14.3	−15.0	−20.0	+8.7	−16.0
Case 2 (%)	+2.9	+15.9	+34.6	+0.5	+19.3	+37.5	+2.9	+11.9	+34.6
Case 3 (%)	−4.7	−11.5	−8.6	−1.9	−14.3	−8.8	−3.4	−15.1	−8.6
Case 4 (%)	+1.2	+4.4	+2.5	+0.3	+5.0	+3.8	+0.5	+2.4	+2.5

**Table 3:** Summary of indices of the flow waveform: the peak arterial flow ($Q_{\mathrm{peak}}$), the time when flow peaks ($t_{\mathrm{peak}}$), and the maximum backflow ($Q_{\max-\mathrm{back}}$), recorded at mid-ascending aorta, mid-femoral and mid-brachial arteries.

Position	Mid-aorta			Mid-femoral			Mid-brachial		
Indices	$t_{\mathrm{peak}}$	$Q_{\mathrm{peak}}$	$Q_{\max-\mathrm{back}}$	$t_{\mathrm{peak}}$	$Q_{\mathrm{peak}}$	$Q_{\max-\mathrm{back}}$	$t_{\mathrm{peak}}$	$Q_{\mathrm{peak}}$	$Q_{\max-\mathrm{back}}$
Unit	s	mL s^−1^	mL s^−1^	s	mL s^−1^	mL s^−1^	s	mL s^−1^	mL s^−1^
**Baseline**	**0.096**	**545**	−**14**	**0.259**	**35**	−**5**	**0.134**	**18.3**	−**2.7**
Case 1 (%)	+1.0	−10.5	−100.0	−17.0	−8.6	−34.0	−10.4	−15.3	−66.7
Case 2 (%)	−10.4	−6.1	+30.7	+0.8	+8.6	+118.0	−0.7	+1.6	+44.4
Case 3 (%)	−4.2	−25.5	+16.4	−0.4	−28.6	−24.0	−0.7	−26.2	+0.0
Case 4 (%)	−3.1	+15.0	+47.1	−0.8	+20.0	+28.0	−0.7	+16.9	+37.0

**Table 4:** Comparison of the peak pressure ($P_{\mathrm{peak}}$) and peak flow rate ($Q_{\mathrm{peak}}$) between the coupled model and the isolated SA model for Cases 1 and 2, estimated at the mid-ascending aorta, mid-femoral and mid-brachial arteries.

Position	Mid-aorta		Mid-femoral		Mid-brachial	
Indices	$P_{\mathrm{peak}}$	$Q_{\mathrm{peak}}$	$P_{\mathrm{peak}}$	$Q_{\mathrm{peak}}$	$P_{\mathrm{peak}}$	$Q_{\mathrm{peak}}$
Units	mmHg	mL s^−1^	mmHg	mL s^−1^	mmHg	mL s^−1^
Case 1	127.2	488.5	136.3	32.3	137.2	15.5
Case 1_iso (%)	+5.6	+7.5	+6.5	+10.8	+4.1	+19.4
Case 2	131.5	512.5	142.4	38.0	141.4	18.6
Case 2_iso (%)	+3.9	+8.3	+2.9	+10.5	+5.6	+3.8

## Appendix A Supplementary data

Application 1
