# Supplementary material for: Study of cardiovascular function using a coupled left ventricle and systemic circulation model
Source: J Biomech. 2016 Aug 16;49(12):2445–54. doi: 10.1016/j.jbiomech.2016.03.009 (PMC5038162; doi:10.1016/j.jbiomech.2016.03.009)
Supplement: Application 1 [file mmc1.pdf]

Appendix I

The Holzapfel–Ogden strain energy function is (Holzapfel and Ogden, 2009)

$$W = \frac{a}{2b}\exp[b(I_1-3)] + \sum_{i=\mathrm{f,s}} \frac{a_i}{2b_i}\{\exp[b_i(I_{4i}^{\star}-1)^2]-1\} + \frac{a_{\mathrm{fs}}}{2b_{\mathrm{fs}}}\{\exp[b_{\mathrm{fs}}(I_{8\mathrm{fs}})^2]-1\}, \tag{12}$$

in which a, b, a_i, and b_i (i = f, s, and fs) are material parameters, f, s and fs indicate the fibre, sheet and normal directions ($\mathbf{f}, \mathbf{s}, \mathbf{n}$), and I_i are the corresponding invariants. The first term of (12) indicate the the matrix property, the second terms represent the fibre and sheet properties, and the last term is due to the coupling between the fibre and sheet directions. From (12) we can derive σ^p

$$\sigma^p = \frac{1}{\det(\mathbb{F})}\left[\frac{\partial W}{\partial \mathbb{F}}\mathbb{F}^T - a\exp(b(I_1-3)) + \beta_{\mathrm{s}}\log(\det(\mathbb{F})^2)\right], \tag{13}$$

(Gao et al., 2014b). The pressure-like term $a\exp(b(I_1-3))/\det(\mathbb{F})$ in (13) ensures that when $\mathbb{F} = \mathbb{I}$, $\sigma^p = 0$. The last term is used to reinforce structure incompressibility. Incompressibility is enforced globally in the Eulerian equation (2), but the interpolation of the Eulerian velocity field to the solid region may not always give a divergence-free discrete Lagrangian velocity field in the structure region. Our previous study showed that the additional constraint $\beta_{\mathrm{s}}\log(\det(\mathbb{F})^2)/\det(\mathbb{F})$ can yield more accurate stress fields (Gao et al., 2014b) with $\beta_{\mathrm{s}} = 500\mathrm{kPa}$.

Appendix II: List of parameter values used for the baseline case

Name	Parameter	Value
Length of period (SA)	T_{SA} (s)	0.9
Density of blood	ρ ($\mathrm{g\,cm^{-3}}$)	1.06
Blood viscosity	μ ($\mathrm{g\ cm^{-1}s^{-1}}$)	0.049
Minimum vessel radius	$r_{\min}$ (μm)	100
External pressure	P_0 (mmHg)	60
Coefficients	k_1 ($\mathrm{g\,s^{-2}\,cm^{-1}}$)	2.00×10^7
of Eh/r_0	k_2 ($\mathrm{cm^{-1}}$)	-22.53
in (9) (Olufsen et al., 2000)	k_3 ($\mathrm{g\,s^{-2}\,cm^{-1}}$)	4.65×10^5
Radius exponent (Olufsen et al., 2000)	ξ	2.76
Asymmetry ratio (Olufsen et al., 2000)	γ	0.405
Penalty coefficient (Gao et al., 2014b)	β_{s} (kPa)	500
End-of-diastolic pressure	$P_{\text{ED-LV}}$ (mmHg)	8
Active tension scaling factor	T_0	3
Material	a (kPa)	0.19
parameters	b	5.08
for the Holzapfel-Ogden	a_{f} (kPa)	1.2
strain energy	b_{f}	4.15
function	a_{s} (kPa)	0.7
in (12)	b_{s}	1.6
(Gao et al., 2014b)	a_{fs} (kPa)	0.24
	b_{fs}	1.3

Appendix III: Summary of the boundary conditions

The boundary conditions for the coupled model are summarized in Fig.1, and detailed below.

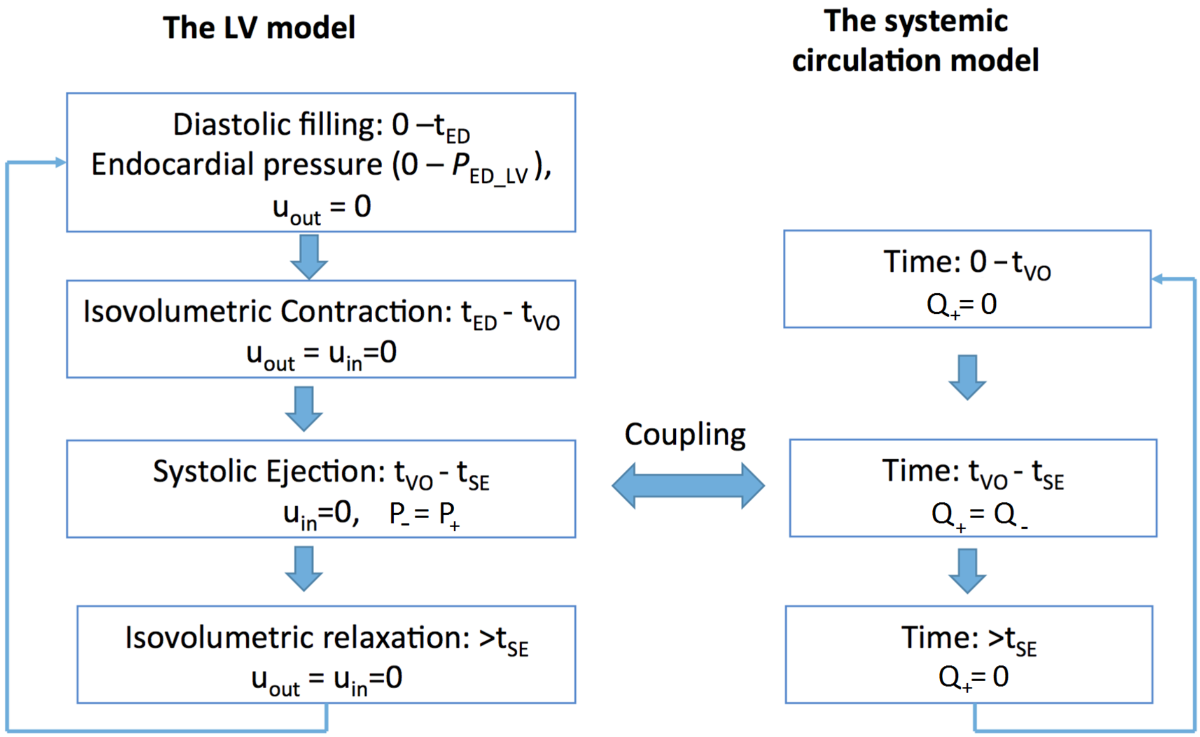

Figure 1: Diagram of applied boundary conditions in a cardiac cycle for the coupled LV-SA model.

- **Diastolic filling:** for $t = 0$ to t_{ED}, where t_{ED} is set to be 0.6 s, the mitral valve is open and a linearly ramped pressure, from 0 to $P_{\text{ED-LV}}$ is applied to the endocardial surface of the LV, $P_{\text{EDLV}} = 8$ mmHg, which is a population-average value. We set $\mathbf{u}_{\text{out}} = 0$ at the outlet of the outflow tract.

- **Isovolumetric contraction:** both valves are closed so at the inlet and outlet $\mathbf{u}_{\text{out}} = \mathbf{u}_{\text{in}} = 0$ for $t = t_{\text{ED}}$ to t_{VO}, where t_{VO} is the time when the AV opens. t_{VO} is determined using the pressure condition: $P_{-} \geq P_{+}$, where $P_{-} = \frac{1}{|\Gamma_a|}\int_{\Gamma_a} p\, dA$, and P_{+} is initially set to be 85 mmHg.

- **Systolic ejection:** we set $P_{-} = P_{+}$ in the outlet of the LV model and $Q_{+} = Q_{-}$ in the SA model in the interface Γ_a as shown in Fig. 3, and $\mathbf{u}_{\text{in}} = 0$ for $t = t_{\text{VO}}$ to t_{SE}, where t_{SE} is the

time when the systolic ejection phase ends, i.e. when the flow rate in the outflow tract in the LV model reduces to zero.

- **Isovolumetric relaxation:** both valves are closed, so $\mathbf{u}_{\text{out}} = \mathbf{u}_{\text{in}} = 0$ in the LV model.
